# The Attitude of Patients from a Romanian Tertiary Cardiology Center Regarding Participation in Biomarker-Based Clinical Trials

**DOI:** 10.3390/medicina57111180

**Published:** 2021-10-31

**Authors:** Iulia Rusu, Nicoleta-Monica Popa-Fotea, Mihaela Octavia Stanculescu, Diana Rusu, Alexandra Dumitru, Alexandru Scafa-Udriste, Oana-Maria Udrea, Miruna Mihaela Micheu

**Affiliations:** 1 Department of Cardiology, Clinical Emergency Hospital of Bucharest, 014461 Bucharest, Romania; iuliarusu015@gmail.com (I.R.); fotea.nicoleta@yahoo.com (N.-M.P.-F.); mihaela.octavia@yahoo.com (M.O.S.); alice.a.dumitru@gmail.com (A.D.); alexscafa@yahoo.com (A.S.-U.); 2 Department of Cardiology, “Carol Davila” University of Medicine and Pharmacy, 050474 Bucharest, Romania; 3 Department of Radiology, “Carol Davila” Central Military Emergency University Hospital, 010825 Bucharest, Romania; diana.rusu995@gmail.com; 4 Department of Psychology, “Grigore Alexandrescu” Clinical Hospital of Bucharest, 011743 Bucharest, Romania; oana_udrea@ymail.com

**Keywords:** biomarker, informed consent, clinical trial, questionnaire

## Abstract

*Background and Objectives*: biomarker-based studies are the cornerstone of precision medicine, providing key data for tailored medical care. Enrollment of the planned number of patients is a critical determinant of a successful clinical trial. Moreover, for inclusive medical care, patients from different socio-demographic backgrounds must be recruited. Still, a significant number of trials fail to reach these prerequisites. Designing the informed consent forms based on the patients’ feedback could optimize accrual. We aimed to explore the attitudes of patients from a Romanian tertiary cardiology center towards participation in biomarker-based clinical trials. *Materials and Methods*: three hundred forty inpatients were interviewed based on a semi-structured questionnaire which included four sections: demographics, personal medical history, attitudes and trust. *Results*: Roughly, 62.5% of the respondents were interested in enrolling, while altruistic reasons were the most frequently expressed. Clear exposure of the possible risks was most valued (37.78%), followed by the possibility of directly communicating with the research team (23.78%). The most frequently chosen answer by acutely ill patients was improvement of their health, whereas chronically ill individuals indicated the possibility of withdrawal without affecting the quality of medical care. Importantly, the participation rate could be improved if the invitation to enrollment were made by both the current physician and the study coordinator (*p* = 0.0001). The level of trust in researchers was high in more than 50% of the respondents, and was correlated with therapeutic compliance and with the desire to join a biomarker study. *Conclusions:* the information gained will facilitate a tailored approach to patient enrollment in future biomarker-based studies in our clinic.

## 1. Introduction

Decoding the patient’s omic data is essential to aiding the successful completion of targeted therapy in clinical practice. Participation in clinical trials is needed in order to guarantee ongoing improvements in patient-specific therapeutic interventions. The role of biomarkers is increasingly promising, considering the continuous development of new targeted therapies. It is generally accepted that well-designed studies including a large number of participants are needed for biomarker-based research validation. However, one of the challenges faced with when conducting a clinical trial is recruitment of the targeted number of participants. The informed consent form (ICF) is ethically and legally required before enrollment in a clinical trial, and it should be tailored to respond to the patients’ needs and values. Full understanding of the ICF is crucial for participation in clinical studies [1]. Besides comprehension of the ICF, there are other determinants of involvement, such as voluntary participation, data protection, data use and participants’ trust in researchers [2,3,4,5]. There is wide interest in improving the design of ICF based on patients’ preferences. Extensive research is needed for the study of patients’ attitude towards participation in clinical trials. Therefore, this observational study aims to explore the attitude of patients from a Romanian tertiary cardiology center regarding involvement in biomarker-based clinical trials.

## 2. Material and Methods

A questionnaire was distributed by trained research staff to evaluate patients’ willingness, attitude and expectations concerning partaking in biomarker-based studies, as well as the general trust in researches, based on a review of literature. The survey methodology has been detailed elsewhere [6]. Briefly, several multidisciplinary consensus meetings between two senior cardiologists and one senior psychologist were organized to assemble and refine the final questionnaire. The study questionnaire c comprised four sections: section A, demographics (7 items); section B, personal disease history and quality of life (7 items); Section C, attitudes and expectations from a biomarker study (9 items) and section D, trust in physicians (4 items) (Supplementary Materials).

### 2.1. Data Collection

The study was approved by the Ethics Committee of the Clinical Emergency Hospital of Bucharest, and was performed in compliance with the principles of the Declaration of Helsinki (approval 5385/25 May 2018). Written informed consent was obtained from all subjects. The final questionnaire was distributed to 340 subjects from the department of cardiology at the Emergency Clinical Hospital. The participants were approached for enrollment during their hospitalization for cardiovascular diseases. ICFs were signed on a separate form than the questionnaire as to ensure the anonymity of the subjects. The participants’ diagnoses were filled in from the electronic medical records for accuracy.

### 2.2. Data Analysis

The data are presented as mean ± standard deviation for continuous variables, or as number and percentage for categorical or nominal data.

The following independent variables: gender, education, age, race, religion, previous hospitalizations in the last 12 months, chronic disease status, previous study enrollment and compliance to treatment, were examined for their effects on the respondents’ answers with the Chi-square test. Statistical analysis was performed with SPSS version 23.

## 3. Results

### 3.1. Respondent Characteristics

The study included 340 subjects, with a mean age of 59.68 ± 13.15 years, predominantly males (70.4%), from an urban area (72.7%), who had some college education or more (84.8%) (Table 1). Only 37 (10.09%) of the respondents had previously participated in a medical trial, and 135 (39.9%) reported a chronic disease. From the subjects requiring medical treatment, 61 (17.9%) were not compliant to the prescribed drugs. The majority of the subjects (81.17%) reported a quality of life ranked at 5 or above on a scale from 1 to 10.

### 3.2. Attitudes and Expectations

A percentage of 85.3% of the participants considered biomarker-based studies to be beneficial, and 62.5% were interested in participating.

The main reason for accepting to take part in such a study was the contribution to research in general (26.1%), followed by the desire to help other subjects with the same disease (21.2%), or for oneself (16.4%), as the information could help one’s own treatment, whereas 15.2% would not participate (Table 2). For the majority of subjects, the paramount reason that would convince them to enroll in such a study would be the possibility to find out its results and the impact on their health (35.16%), followed at large distance by the certainty that participation will improve their health status in the future (15.24%). The certainty that their rights would be respected during the study was considered important by 10.57%, while others reported as essential the possibility to retire from the trial at any time without affecting the quality of current/future medical treatment (8.54%). Other considerations referred to the use of the biological samples (6.3%), material compensations (5.69%) and the non-invasive nature of the study (4.67%) (Table 3). For those agreeing to participate, clear exposure of the possible risks is foremost valued (37.78%), followed by the possibility of directly communicating with the research team (23.78%). Other aspects, such as the study scope being extensively explained in clear terms (19.78%), statistics about the biomarkers’ importance (8.67%) or drawings, schemas, and tables containing relevant data (2.44%), were only marginally considered (Table 4). Long and complicated phrases were futile to convince participants (46.22%), as well as insufficient data about individual rights (23.76%) or medical data in general (16.85%) or too-detailed medical information (12.96%) (Table 5).

### 3.3. Trust in Physicians

**Figure 1:**
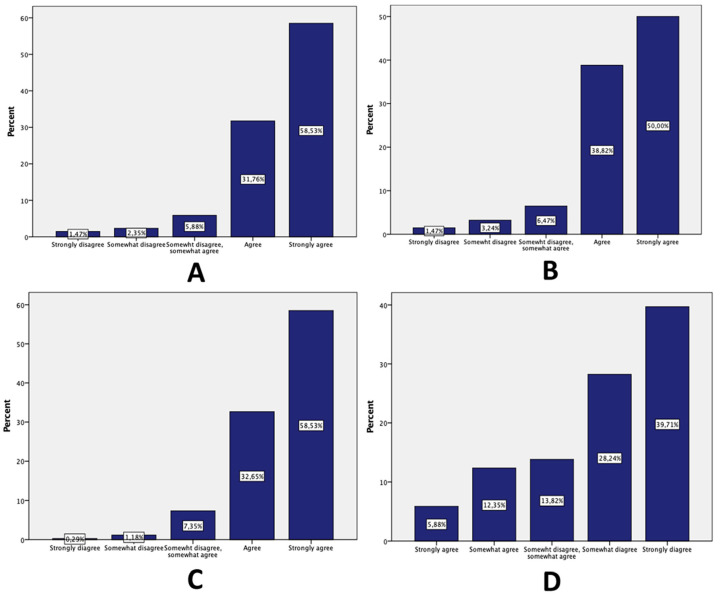
Participants’ responses assessing levels of trust in medical researchers. (**A**) Physicians involved in research care only about the best for each patient. (**B**) Physicians tell their patients all the information they need to know about the study. (**C**) I completely trust doctors involved in medical research. (**D**) Physicians involved in research treat patients like “guinea pigs.”

The majority of the respondents trusted medical researchers (58.4% strongly agreed), and believed that physicians involved in research only care about the best for each patient (strongly agreed, 58.53%) and would disclose all the information needed to be known about the study (58.53%) (Figure 1). Neearly 67% of the participants somewhat or strongly disagreed that doctors involved in research treat patients like “guinea pigs”.

### 3.4. Respondent Characteristics, Questionnaire Resolutions and Significant Associations

Participants with chronic diseases were more likely to adhere to prescribed medical treatment than those with acute illnesses (*p* = 0.001) and had more frequently been involved in previous medical studies (*p* = 0.003). Interestingly, chronic illnesses did not significantly affect the quality of life (*p* = 0.636), perception about biomarker studies (*p* = 0.98), interest (*p* = 0.317) or motivation to engage in such trials (*p* = 0.69).

The level of education did not influence compliance to treatment (*p* = 0.302), access to medical studies (*p* = 0.613), quality of life (*p* = 0.277) or the reasons to participate (*p* = 0.124), but instead influenced the interest to involve (*p* = 0.033), even if the correlation was weak (rho = 0.117) or the information considered important (*p* = 0.029). Those with higher educations were less interested in participating and considered essential the clear exposure of the possible risks derived from the study, but with a weak correlation (rho = 0.12). Interestingly, participants with some higher education saw the questionnaire more positively compared with those with 4-year degree (*p* = 0.0001).

The heart disease for which participants were hospitalized did not influence participation in previous medical studies (*p* = 0.065). Instead it was observed that subjects who presented with acute conditions (acute coronary syndromes, acute pulmonary edema) had a tendency not to be compliant to treatment (*p* = 0.011), unlike those with chronic conditions (chronic heart failure); the type of cardiovascular disease for which the subject was hospitalized did not influence the quality of life (*p* = 0.821). Instead, the information that convinced participants to enroll was different and significant (*p* = 0.046); for those with acute conditions (acute myocardial infarction), participation in the study could substantially improve their health in the future, while for those with chronic conditions (chronic heart failure, atrial fibrillation, arterial hypertension), the possibility to withdraw from the study at any time without affecting the quality of the medical treatment and the assurance that their rights will be respected during the study were more valued.

The level of trust in doctors was not influenced by sex, age, religion, community size, education, ethnicity or marital status, while the interest in participating in a clinical study on biomarkers was higher in those with high levels of trust in physicians and those that had already been enrolled in another study. Trust in physicians was also correlated with compliance (*p* = 0.046, rho = −0.107). Those that strongly agreed that physicians involved in research wanted only the best for each patient (*p* = 0.014) and that all the required information is revealed (*p* = 0.018) were more prone to participate. Of note, the participation rate in the study could be improved if the invitation to enroll were to be made by both the current physician and the study coordinator (*p* = 0.0001).

## 4. Discussions

Biomarkers are the keystone of biomedical research and daily clinical practice, and patients’ involvement in biomarker-based clinical trials is essential to achieving precision medicine. Subsequently, extensive research has been conducted worldwide to explore the patients motivation and willingness to participate in such studies [4,7,8,9,10,11]. The subject’s acceptance was driven by many intimate impulses that should be addressed in order to increase participation. The arguments required to motivate engagement should surpass the meaningless definitions of being good or indicated. Analytical insights and strategies in biomarkers studies have been questioned in some research studies [12]. However, there is no data about the attitude of Romanian patients towards participation in biomarker-based research. To our knowledge, this is the first study addressing this gap in the literature.

Roughly 60% of the respondents were interested in such research, and their willingness was correlated with the level of trust in physicians. Importantly, if the consent to enroll is requested by both the current physician and the coordinator of the study, the participation rate increases, raising the awareness that a collaboration between the research team and the clinicians could increase the inclusion rate, rather than the general trends that call for patients through funding polices [13].

The possibility of finding out the study results is another important aspect. A Swedish study showed that participants considered the possibility to influence the use of data and also data availability most important, rather than respected rights [14], which is in accordance with our findings (35.16% were interested in the possibility of finding out the study results). Regarding this particular aspect, implementation of a digital consent system would give participants the opportunity to be informed about the usage of their data and the results of the research to which their data have contributed [15].

Comprehension of the ICF poses problems for many participants. Studies have showed that an important segment of the participants do not fully understand all the informed consent documents, because of the difficulty of the material and its legalistic wording [16,17]. Making the ICF briefer could improve patients’ comprehension [18]. In our study, the improvement of the comprehension by clear exposure, drawings, schemas or tables with relevant data of the study was not substantially demanded by participants. Instead, the priority interest was clear exposure of the possible risks.

Also, the information must be adapted according to the clinical situation of the subject; on the one hand we saw that subjects with acute illnesses were rather interested if the study could improve their health in the future, on the other hand those with chronic conditions, already acknowledging their disease, valued the possibility to withdraw from the study at any time without affecting the quality of the medical treatment and the assurance that their rights will be respected during the study more.

The level of trust in researchers was high in more than 50% of the respondents, much stronger compared with other studies [19], and was correlated with therapeutic compliance and with the desire to engage in a biomarker study.

## 5. Conclusions

Our survey data analysis offered an in-depth understanding of patients’ perceptions, thus empowering a tailored approach to patient enrollment in future biomarker-based studies in our clinic. Depending on the characteristics of the studies carried out (addressing either acute or chronic cardiovascular conditions), the informed consent forms will be revised so as to provide the necessary information to that respective category of patients. Moreover, our results can be applied in other clinics to can refine the design of ICF according to local characteristics (such as the predominance of acute or chronic cases).

## Figures and Tables

**Table 1:** Study population characteristics (*N* = 340).

Variables	Number (Percentage) of Patients
Age (years)	59.68 ± 13.15 (19–92)
Sex, female, *n* (%)	101 (29.6%)
Community size	
Urban area	248 (72.7%)
Rural area	90 (26.4%)
Education	
Graduate degree	94 (27.6%)
High school graduate	85 (24.95)
Some college	108 (31.7%)
4-year degree	52 (15.2%)
Ethnicity	
European	314 (92.1%)
Indo-European	10 (2.9%)
Indian	1 (0.3%)
Mongol	1 (0.3%)
Married/partner	250 (73.3%)
Religion	
Orthodox	308 (90.3%)
Catholic	3 (0.9%)
Hindus	1 (0.3%)
Pentecostal	3 (0.9%)
Baptist	1 (0.3%)
Muslim	1 (0.3%)
Atheist	5 (1.5%)
Chronic disease	
Yes, cardiovascular	127 (37.35)
Yes, other	8 (2.35%)
No	205 (60.29%)
Compliance to treatment	
Yes	61 (17.9%)
No	219 (64.4%)
Not the case	60 (17.65%)

**Table 2:** Primary reasons for agreeing to enroll in a biomarker study (*N* = 340).

Reasons	Number (Percentage) of Patients
To help other patients with the same disease as me	72 (21.1%)
To help other members of my family that could have the same disease	29 (8.5%)
For myself-maybe the information obtained would be helpful	56 (16.4%)
For contributing to the enrichment of the disease’s knowledge, even if would not be direct beneficiary	32 (9.4%)
For contributing to science in general	8 (26.1%)
I would not participate	52 (15.2%)
1&2	4 (1.2)
1&5	3 (0.9%)
3&4	1 (0.3%)
3&5	1 (0.3%)
4&5	1 (0.3%)

**Table 3:** Important information to convince the respondent to participate in a biomarker study (Choose one or two options).

Information	Number (Percentage) of Patients
The possibility to find out the results of the study and their impact on my health	173 (35.16%)
The non-invasive nature of sampling	23 (4.67%)
Material compensations	28(5.69%)
The knowing of the biological sample usage	18 (3.66%)
The exposure of the advantages if enrolling	17 (3.45%)
The certainty that participation in the study would ameliorate my health status	75 (15.24%)
The certainty that samples will not be used for purposes other than those mentioned	31 (6.3%)
The certainty that my rights would be respected throughout the study	52 (10.57%)
The possibility to retire from the study at any time without affecting the quality of the medical treatment	42 (8.54%)
Others	3(0.61%)
Nothing	30 (6.1%)

**Table 4:** Important aspects for those involved in a biomarker study (Choose one or two options).

Aspects	Number (Percentage) of Patients
Clear exposure of the possible risks	170 (37.78%)
Explanation of study purpose in accessible terms	89 (19.78%)
Drawings, schemas, tables with relevant data	11 (2.44%)
Statistics on biomarkers’ importance	39 (8.67%)
Direct communication with the research team	107 (23.78%)
Others	1 (0.22%)
Nothing	33 (7.33%)

**Table 5:** The useless aspects that the physician could expose in the study presentation.

Aspects	Number (Percentage) of Patients
Long and complicated phrases/the lack of accessible terms	214 (46.22%)
Insufficient medical data	78 (16.85%)
Too detailed medical information	60 (12.96%)
Insufficient data about personal rights	110 (23.76%)
Others	1 (0.21%)

## Data Availability

Not applicable.

## Supplementary Materials

The following is available online at https://www.mdpi.com/article/10.3390/medicina57111180/s1: Questionnaire on the attitude of patients admitted in Clinical Emergency Hospital of Bucharest towards the participation in biomarker-based clinical trials.
