# Supplementary material for: The Attitude of Patients from a Romanian Tertiary Cardiology Center Regarding Participation in Biomarker-Based Clinical Trials
Source: Medicina (Kaunas). 2021 Oct 31;57(11):1180. doi: 10.3390/medicina57111180 (PMC8625162; doi:10.3390/medicina57111180)
Supplement: Supplementary file 1 [file medicina-57-01180-s001.zip › medicina-1442553-supplementary.pdf]

Questionnaire on the attitude of patients admitted in Clinical Emergency Hospital of Bucharest towards the participation in biomarker-based clinical trials

1. Identification code (*to be completed by the investigator in order of recruitment*): Q-

2. Age:

3. Sex:
   - ☐ M
   - ☐ F

4. Telephone number (optional):

5. Residence:
   - ☐ Urban
   - ☐ Rural

6. Education:
   - ☐ Higher education
   - ☐ Secondary education
   - ☐ High school
   - ☐ Primary school/Gymnasium
   - ☐ No degree

7. Ethnicity:
   - ☐ ………………….
   - ☐ I don’t know/I don’t answer

8. Religion/ Faith:
   - ☐ ………………….
   - ☐ I don’t know/I don’t answer

9. Marital status:
   - ☐ Married
   - ☐ Single
   - ☐ Divorced/separated
   - ☐ Widow
   - ☐ I don’t answer

10. Heart disease (admission diagnosis)*: ………..……………………………………………………

11. The onset of the disease*:……………………………………………….…………………………….

**to be completed by the investigator according to medical chart*

12. Any condition for which you have been hospitalized in the last 12 months?

- ☐ Yes, cardiovascular disease
- ☐ Yes, another condition………………………………………………………………………
- ☐ No

13. Are you diagnosed with a chronic illness?

- ☐ Yes, cardiovascular disease
- ☐ Yes, cancer
- ☐ Yes, another condition..……………………………………………………………..
- ☐ No

14. Are you taking medication as prescribed?

- ☐ Yes
- ☐ No
- ☐ It is not the case

15. Have you ever participated in a medical study?

- ☐ Yes
- ☐ No

16. Impact of the illness on quality of life – on a scale of 1 to 10 (1 being deeply altered quality of life and 10 being excellent quality of life). How would you grade your quality of life at the present moment?**

1 2 3 4 5 6 7 8 9 10

***by quality of life we define the perceived physical, mental and social well-being*

17. Do you consider valuable a clinical study using biomarkers?

- ☐ Yes
- ☐ No
- ☐ Impartial

In the next section we refer to the hypothetical situation that you will be asked to participate in a biomarker-based clinical trial (for example: blood products, tissue or blood cells, genes/DNA).

18. Are you interested in participating in this study?

- ☐ Yes
- ☐ No
- ☐ Impartial

19. What is the main reason for accepting to participate in this study?

- ☐ Helping other patients who have the same disease as I have
- ☐ Helping my family members that could have the same disease as I have in the future
- ☐ Maybe the resulting information will be helpful for me
- ☐ In order to contribute to acquisition of knowledge about my disease, even if my family and I would not have any direct benefits
- ☐ In order to contribute to research
- ☐ Another reason:………………………………………………………………………………
- ☐ I would not participate

20. What information do you consider important in order to convince you to participate in this study? *Check two options*

- ☐ The possibility of finding out the study results and their impact on my health
- ☐ A non-invasive sample collection for the study
- ☐ Financial compensation for participants
- ☐ Knowing how the samples collected will be used
- ☐ Knowing what are the benefits of participating in the study
- ☐ The certainty of improving my well-being in the future as a result of participating in the study
- ☐ Ensuring that my samples will not be used in other ways than those mentioned
- ☐ Ensuring that my rights will be respected during the whole study
- ☐ The possibility of withdrawal from the study without affecting the quality of medical services
- ☐ Other:
- ☐ Nothing

21. If you would participate in this study, which of the following items do you find important for you? *Check two options*

- ☐ To be clearly stated the potential risks that I am exposed to
- ☐ The purpose of the study explained at length, in easy terms
- ☐ Drawings, schemes, tables with relevant data

☐ Statistics regarding the importance of biomarkers

☐ The possibility to communicate directly with the research team

☐ Other:…………………………………………………………………………....

☐ Nothing

22. What do you consider not useful in the explanations provided by the doctor when your participation agreement is requested? *Check two options*

☐ Long and complicated sentences /the lack of explanations in easy terms

☐ Insufficient medical information

☐ Too much medical information

☐ Insufficient information regarding your rights (the possibility of withdrawal from the study without affecting the quality of the medical services)

☐ Other:………………………………………………………………………….

23. Your trust or your participation in the study would be increased if the participation agreement would be asked by:

☐ Your treating doctor (the doctor who is treating your current condition)

☐ The clinical study coordinator

☐ Both the treating doctor and the clinical study coordinator

☐ Any doctor involved in the study

☐ Under no circumstances

24. Do you consider useful this questionnaire? How would you grade it on a scale of 1 to 5 (1 being not useful at all and 5 being very useful).

1 2 3 4 5

25. Under which of the following circumstances would you agree to be contacted?

☐ For completing the information from this questionnaire

☐ For other studies conducted at Clinical Emergency Hospital of Bucharest, Cardiology Departament

☐ Under no circumstances

Please tell us what is your opinion regarding the following statements:

26. The doctors involved in research wish only the well-being of each patient.

☐ I completely disagree

☐ I disagree

- ☐ Neutral/indifferent
- ☐ I agree
- ☐ I completely agree

27. The doctors provide the patients all the information/all it is necessary to know about the study.
    - ☐ I completely disagree
    - ☐ I disagree
    - ☐ Neutral/indifferent
    - ☐ I agree
    - ☐ I completely agree

28. I completely trust the doctors who are involved in medical research
    - ☐ I completely disagree
    - ☐ I disagree
    - ☐ Neutral/indifferent
    - ☐ I agree
    - ☐ I completely agree

29. The doctors involved in research treat the patients as “objects”.
    - ☐ I completely agree
    - ☐ I agree
    - ☐ Neutral/indifferent
    - ☐ I disagree
    - ☐ I completely disagree

30. Is there anything else you would like to add for this questionnaire (comments, sugestions)?

………

………

Thank you for the participation,
QUEST team.
